# Moroccan and Pakistani women’s knowledge and perceptions on cervical cancer screening and HPV self-sampling acceptability in Catalonia, Spain: a mixed-methods study

**DOI:** 10.1186/s12913-025-13488-w

**Published:** 2025-11-20

**Authors:** Jone G. Lurgain, Paula Peremiquel-Trillas, Hakima Ouaarab-Essadek, Khadija Mellouki, Sumaira Malik-Hameed, Andleed Sarif, Valentina Rangel-Sarmiento, Laia Bruni, Guy Harling, Clare Gilham

**Affiliations:** 1 Department of Public Health, Environments and Society, Faculty of Public Health and Policy, London School of Hygiene and Tropical Medicine, Keppel Street, London, WC1E 7HT UK; 2 Cancer Epidemiology Research Programme, Catalan Institute of Oncology, Av Gran Via 199-203, L’Hospitalet de Llobregat, Barcelona, 08908 Spain; 3 Bellvitge Biomedical Research Institute – IDIBELL, Av Gran Via 199-203, L’Hospitalet de Llobregat, Barcelona, 08908 Spain; 4 Consortium for Biomedical Research in Epidemiology and Public Health–CIBERESP, Carlos III Institute of Health, Av De Monforte de Lemos 5, Madrid, 28029 Spain; 5 Faculty of Nursing and Health Sciences, University of Barcelona, C/Feixa Llarga s/n, L’Hospitalet de Llobregat, Barcelona, 08907 Spain; 6 Community & Public Health Team (ESPIC), Centre for International Health and Infectious Diseases, Drassanes-Vall d’Hebron, Carrer de Sant Oleguer, 17, Barcelona, 08001 Spain; 7 Institute for Global Health, University College London, London, UK; 8 Africa Health Research Institute, KwaZulu-Natal, Durban, South Africa; 9 School of Nursing & Public Health, University of KwaZulu-Natal, Durban, South Africa; 10 MRC/Wits Rural Public Health & Health Transitions Research Unit, University of Witwatersrand, Johannesburg, South Africa; 11 Department of Non-communicable Disease Epidemiology, Faculty of Epidemiology and Population Health, London School of Hygiene and Tropical Medicine, Keppel Street, London, WC1E 7HT UK

**Keywords:** Cervical cancer, Human papillomavirus, HPV screening, HPV self-sampling, HPV self-collection, Migrants, Spain

## Abstract

**Background:**

Disparities in cervical cancer (CC) screening participation persist, with lower rates among immigrant women from low-resource countries compared to native European women. Evidence-based strategies to reach under-screened women are thus needed, such as adopting self-sampling for human papillomavirus (HPV) testing. Studies have demonstrated that women are receptive to HPV self-sampling. However, results may not be generalizable to all ethnic groups and settings. This is the first study in Spain assessing HPV self-sampling acceptability among immigrant populations. A mixed-methods study was used to explore knowledge and perceptions of CC screening and attitudes towards HPV self-sampling among Moroccan and Pakistani women in Catalonia.

**Methods:**

Eight focus group discussions and twenty-two semi-structured interviews were conducted. After a short demonstration of two self-sampling devices, women were invited to try them at home and complete an acceptability survey for each device, including questions assessing screening preferences, perceived self-efficacy, trust in the test results and willingness to use the self-collection method again.

**Results:**

Important barriers, such as lack of information about CC screening, and misconceptions about HPV risk were identified. Overall, Moroccan and Pakistani women expressed a preference for clinician-based screening over self-sampling. Over half of the participants (56%) accepted to try at least one self-sampling device. However, concerns about collecting the sample correctly and distrust in the test result were raised.

**Conclusion:**

Increasing awareness and empowering Moroccan and Pakistani women with culturally appropriate information about the benefits of CC screening is the first step to successfully implement HPV self-sampling. Concerns regarding self-efficacy need also to be addressed before implementing new organised screening programmes using HPV self-sampling in Catalonia, Spain. A peer-based approach using culturally appropriate materials is proposed to best inform, educate, foster confidence, and advocate for the uptake of HPV self-sampling among these two groups of women.

**Supplementary Information:**

The online version contains supplementary material available at 10.1186/s12913-025-13488-w.

## Background

Human papillomavirus (HPV) is the most common sexually transmitted infection (STI): in Western countries it is estimated that 80% of all sexually active unvaccinated individuals will contract an HPV infection within their lifetime [1]. Despite its typical natural course of spontaneous elimination, a persistent infection can result in precancerous lesions which may progress to cervical cancer (CC) after 5 to 20 years [2–4]. Globally, CC affects over 662,000 women and causes nearly 350,000 deaths every year [5].

CC can be effectively prevented through HPV vaccination and organised screening programmes. In Europe, well established population-based organised screening programmes have reduced mortality by 80% or more among screened women [6] and, specifically, HPV-based screening has proven to be more effective than traditional cytology in reducing the incidence of precancerous cervical lesions [7, 8]. Yet disparities in screening participation persist, with lower rates documented among immigrant women from low- and middle-income countries (LMICs) compared to native European women [9–12]. For instance, earlier studies conducted in Spain indicate that Moroccan women rank among the immigrant groups with lowest uptake rates of cervical and breast cancer screening [13–15] and research in different high-income countries (HICs) also suggests that Pakistani women are an under-screened immigrant group [16–18]. Evidence-based strategies to reach unscreened or under-screened women are thus needed, such as the adoption of self-sampling for HPV testing [19–22].

In Spain, CC screening is currently transitioning from opportunistic to population-based programmes. In April 2019, the Ministry of Health [23] urged all autonomous regions to transition to organised population-based CC screening programmes using HPV testing as the primary screening method. Several screening programmes, such as in the Netherlands [24] and Australia [25], offer self-sampling as an alternative screening approach for women who do not wish to attend for clinician-based screening. The new population-based screening programme in Catalonia, Spain [26] will take a further step by designating HPV self-sampling as the primary method of sample collection and will be offered to all women between the ages of 30 and 65. In addition, clinician-based screening may be requested for those who prefer. Eligible women will be invited through short-message service (SMS) to collect a self-sample device from a near-by pharmacy. Pharmacies will collect and deliver the samples to the laboratories and results will be delivered through the Catalan digital personal health App (La Meva Salut) [27].

To date, diagnostic accuracy studies support HPV self-sampling, demonstrating comparable specificity and sensitivity to clinician-based samples when using PCR technology [28–30]. Studies conducted in both HICs and LMICs have shown varying preferences of HPV self-sampling over clinician-based sampling [18, 31–34] and a wide range of factors influencing these preferences [10, 24, 35–38]. Individual characteristics, such as age and education, influence screening preferences [36, 37]. Also, psychological, social, cultural and health system determinants shape decisions around whether or not to get screened and which screening method they choose [10, 35]. For instance, study participants emphasize the potential of HPV self-sampling to overcome common barriers to conventional screening such as time constraints, embarrassment and discomfort [24, 35], but also mention challenges regarding self-sampling, such as the correct execution of the procedure and the trust in the result [24, 35, 38]. Therefore, assessing acceptability of HPV self-sampling to women is crucial before implementing this sampling method for CC screening.

Although there is substantial qualitative evidence regarding acceptability of HPV self-sampling from some regions, such as sub-Saharan Africa (SSA) [35], most published literature on HPV self-sampling relies on data from quantitative questionnaires. Women are generally receptive to HPV self-sampling [31, 33, 36], yet results from these studies may not be generalizable to all ethnic groups and settings [39]. Specifically in Spain, research into HPV self-sampling has been limited to a few quantitative studies assessing acceptability among native women who attend regular CC screening [40–42], with no study exploring HPV self-sampling among migrant populations. To address this gap, we carried out a mixed-methods study to explore the knowledge and perceptions of CC screening and acceptability of HPV self-sampling among Moroccan and Pakistani women residing in Spain.

## Methods

### Study design

A qualitative-driven mixed-methods study was conducted to explore Moroccan and Pakistani women’s knowledge and perceptions of CC screening and assess HPV self-sampling acceptability. The qualitative component included focused group discussions (FGDs) and semi-structured interviews (SSIs), focusing on knowledge, risk perceptions and screening preferences. The quantitative component involved a survey questionnaire, including sociodemographic characteristics, previous CC screening attendance and HPV self-sampling attitudes and experiences. We combined ‘convergence’ and ‘complementary’ triangulation approaches, where both qualitative and quantitative data were collected and analysed separately and then compared for compatibility [43]. The theoretical framework of acceptability proposed by Sekhon et al. (2017) [44] was used as a basis for the analysis of seven constructs: intervention coherence, affective attitudes, self-efficacy, perceived effectiveness, opportunity costs, burden and ethical concerns. Participants are identified by their country of origin and age defined as younger (< 40 years) or older (≥ 40 years).

### Participants and research setting

We recruited first-generation Moroccan (*N* = 36) and Pakistani (*N* = 37) immigrant women aged 24–65 regardless of their CC screening status. We included women below the HPV self-sampling CC screening age (< 30 years) to capture future willingness to participate in the programme. We combined purposive and snowball sampling to recruit participants in socially deprived areas with high concentration of immigrants in the province of Barcelona. Recruitment was done predominantly through Moroccan and Pakistani key informant networks (e.g. religious and community-based associations) and in collaboration with community health workers in Barcelona. Further details about methodology are available elsewhere [45].

### Data collection

#### Focus groups and individual interviews

We conducted 8 FGDs, each comprising 3–8 women and 22 SSIs between September and December 2022. Topics addressed knowledge on CC and screening, risk perceptions, women’s attitudes towards HPV self-sampling and participants’ preferences and ideas for implementing HPV self-sampling and strategies to raise awareness about CC screening (see FGD and SSI topic guides in English in Supplementary material 1 and 2). All FGDs were held in convenient and familiar places for the participants (e.g., mosques or faith-based associations, community centres and health facilities) and were facilitated by two experienced community health providers who shared language and cultural background with participants. The first author (JGL) and two research assistants (RAs) (KM and AS) conducted the SSIs at locations selected by participants (e.g., their homes, interviewers’ home, religious and community centres). Towards the end of the FGDs and SSIs, women were shown two self-sampling devices: a swab (FLOQSwabs^®^, Copan, Italy) and a brush (EvalynBrush^®^, Rovers Medical, The Netherlands), both validated for HPV detection on multiple PCR-based HPV assays [46]. Subsequently, the discussion was focused on the HPV self-sampling and its advantages and disadvantages in the context of CC screening.

#### Self-sampling acceptability survey

All participants completed a short socio-demographic questionnaire, including previous attendance for CC screening and confidence of using HPV self-sampling (Supplementary material 3). All but two participants (one pilot participant, one left the FGD early) were invited to use both self-sampling devices, which included written instructions in Spanish. Women were informed in advance that they were participating in a trial and would not receive their results but were offered the opportunity to schedule a clinician-based CC screening appointment. Those who accepted the self-sampling devices were asked to complete a paper self-administered questionnaire for each self-sampling device, including nineteen questions assessing screening preferences, self-efficacy, trust in the test result, ease of use, safety and receptiveness of each self-sampling device. The questionnaire was translated into Urdu and Arabic, as well as English and Spanish, and was assessed for comprehensiveness by bilingual research team members, a Moroccan university student, a Pakistani university student and two participants. Women who declined to use one or both self-sampling devices were asked to record their reasons (see the acceptability questionnaires in English in Supplementary material 4).

### Data analysis

#### Qualitative data analysis

All FGDs and SSIs were transcribed directly from Darija (Moroccan vernacular Arabic) and Urdu into Spanish and English, respectively. Thematic content analysis [47] was conducted, combining inductive and deductive approaches, to identify themes and sub-themes. After initial familiarization and independent idea generation from first transcripts by the first author (JGL) and another investigator (PPT), potential themes and sub-themes were discussed and a codebook agreed. Transcripts were uploaded into the qualitative software ATLAS.ti 23 [48] for coding. Data from both countries of origin were analysed in parallel.

#### Quantitative data analysis

Quantitative data from questionnaires were collected and managed using REDCap web-based software platform [49, 50] and analysed using STATA 16 [51]. Analysis of sociodemographic and acceptability data was restricted to the women who were invited to try the devices. The quantitative results are presented separately for each device since the acceptability questionnaire was completed for each. The study was not powered to make statistical inferences and no statistical hypothesis testing was pre-planned, however Fishers Exact Test and Person’s Chi-Squared Test were used in an ad-hoc analysis to compare the acceptance rates between country of origin, CC screening status, employment status, and time since migration to Spain. These results therefore should be interpreted with caution.

### Data triangulation

A two-fold data triangulation process was performed: first, results from the FGDs and SSIs were compared to identify patterns of convergence and divergence; second, qualitative and quantitative data were compared for a comprehensive data analysis [52].

### Ethical considerations

The study was approved by the Research Ethics Committees of the London School of Hygiene and Tropical Medicine (26186), Bellvitge University Hospital (PR 140/22) and Vall d’Hebron University Hospital (PR(AG)317/2022). Each participant provided written informed consent prior to data collection and was given a 10-trip public transportation pass as compensation.

## Results

### Participants’ characteristics

Socio-demographic details of all study participants (*N* = 71; Moroccan: *N* = 37, Pakistani: *N* = 34), as well as for those who accepted to try at least one of the self-sampling devices (56%, *N* = 40), are summarized in Table 1. Participants were aged between 24 and 65, with a median age of 40. Most (68%, *N* = 48) were educated beyond secondary school, but only 18% were employed either formally or informally. Most were or had been married (94%) and had children (87%). Three quarters had lived in Spain for at least 5 years, but half needed a translator to a certain degree. Only 6% had never heard of CC screening and 72% had previously been screened. Results regarding participants’ acceptability of HPV self-sampling are described below.

**Table 1:** Socio-demographic characteristics of study participants invited for self-sampling by country of origin (*N* = 71)^1^

	Total				Morocco				Pakistan			
	Invited for self-sampling		Accepted any self-sampling device		Invited for self-sampling		Accepted any self-sampling device		Invited for self-sampling		Accepted any self-sampling device	
	N	(%)^2^	N	(%)^3^	N	(%)^2^	N	(%)^3^	N	(%)^2^	N	(%)^3^
**Participants**	**71**	***100%***	**40**	***56.3***	**34**	***100%***	**24**	***70.6%***	**37**	***100%***	**16**	***43.2%***
**Age median (IQR)**	40	*(34–48)*	40	*(35–46)*	42	*(35–48)*	38	*(34.5–47)*	39	*(33–46)*	40.5	*(37–44)*
**Age groups**												
< 30 years	8	*11.3%*	4	*50.0%*	3	*8.8%*	2	*66.7%*	5	*13.5%*	2	*40.0%*
30–39 years	26	*36.6%*	15	*57.7%*	12	*35.3%*	11	*91.7%*	14	*37.8%*	4	*28.6%*
40–49 years	21	*29.6%*	16	*76.2%*	10	*29.4%*	8	*80.0%*	11	*29.7%*	8	*72.7%*
> 50 years	15	*21.1%*	5	*33.3%*	5	*23.5%*	3	*37.5%*	7	*18.9%*	2	*28.6%*
**Level of studies**												
No studies	8	*11.3%*	3	*37.5%*	6	*17.6%*	3	*50.0%*	2	*5.4%*	0	*0.0%*
Primary school	15	*21.1%*	11	*73.3%*	9	*26.5%*	8	*88.9%*	6	*16.2%*	3	*50.0%*
Secondary school	25	*35.2%*	12	*48.0%*	14	*41.2%*	10	*71.4%*	11	*29.7%*	2	*18.2%*
University or vocational training	23	*32.4%*	14	*60.9%*	5	*14.7%*	3	*60.0%*	18	*48.6%*	11	*29.7%*
**Employment**												
Employed (formal or informal)	13	*18.3%*	10	*76.9%*	11	*32.4%*	9	*81.8%*	2	*5.4%*	1	*50.0%*
Unemployed, housewives or retired	58	*81.76%*	30	*51.7%*	23	*67.7%*	15	*65.2%*	35	*94.6%*	15	*42.9%*
**Marital status**												
Single	4	*5.6%*	2	*50.0%*	4	*11.8%*	2	*50.0%*	0	*0.0%*	0	*0.0%*
Married	58	*81.7%*	32	*55.2%*	24	*70.6%*	18	*75.0%*	34	*91.9%*	14	*41.2%*
Separated, divorced or widowed	9	*12.7%*	6	*66.7%*	6	*17.6%*	4	*66.7%*	3	*8.1%*	2	*66.7%*
**Children**												
Yes	62	*87.3%*	34	*54.8%*	26	*76.5%*	19	*73.1%*	36	*97.3%*	15	*41.7%*
No	9	*12.7%*	6	*66.7%*	8	*23.5%*	5	*62.5%*	1	*2.7%*	1	*100.0%*
**Time since migration to Spain**												
≤ 5 years	18	*25.4%*	10	*55.6%*	10	*29.4%*	8	*80.0%*	8	*21.6%*	2	*25.0%*
6–10 years	19	*26.8%*	10	*52.6%*	4	*11.8%*	3	*75.0%*	15	*40.5%*	7	*46.7%*
> 10 years	34	*47.9%*	20	*58.8%*	20	*58.8%*	13	*65.0%*	14	*37.8%*	7	*50.0%*
**Translator necessity at health centre**												
I always need a translator	18	*25.4%*	10	*55.6%*	7	*20.6%*	6	*85.7%*	11	*29.7%*	4	*36.4%*
Most of the times I need a translator	7	*9.9%*	6	*85.7%*	2	*5.9%*	2	*100.0%*	5	*13.5%*	4	*80.0%*
Sometimes I need a translator	10	*14.1%*	4	*40.0%*	5	*14.7%*	2	*40.0%*	5	*13.5%*	2	*40.0%*
I do not need translator at all	18	*25.4%*	12	*66.7%*	10	*29.4%*	8	*80.0%*	8	*21.6%*	4	*50.0%*
**Self-perception of religiosity**												
Very religious	22	*31.0%*	12	*54.5%*	12	*35.3%*	7	*58.3%*	10	*27.0%*	5	*50.0%*
Little or somehow religious	45	*63.4%*	24	*53.3%*	19	*55.9%*	14	*73.7%*	26	*70.3%*	10	*38.5%*
**Cervical cancer screening status**												
I don’t know what CC screening is	4	*5.6%*	0	*0.0%*	0	*0.0%*	0	*0.0%*	4	*10.8%*	0	*0.0%*
Never screened	14	*19.7%*	10	*71.4%*	7	*20.6%*	6	*85.7%*	7	*18.9%*	4	*57.1%*
Ever screened^4^	51	*71.8%*	29	*56.9%*	25	*73.5%*	17	*68.0%*	26	*70.3%*	12	*46.2%*
**Time since last screening test**												
< 1 year	14	*27.5%*	9	*64.3%*	8	*32.0%*	7	*87.5%*	6	*23.1%*	2	*33.3%*
1–3 years	20	*39.2%*	8	*40.0%*	11	*44.0%*	5	*45.5%*	9	*34.6%*	3	*33.3%*
3–5 years	9	*17.6%*	6	*66.7%*	2	*8.0%*	2	*100.0%*	7	*26.9%*	4	*57.1%*
> 5 years	6	*11.8%*	4	*66.7%*	2	*8.0%*	1	*50.0%*	4	*15.4%*	3	*75.0%*

IQR: Interquartile range

^1^Due to missing data, the total percentages may not add up to 100%

^2^Percentages calculated by column

^3^Percentages calculated by row

^4^Two participants did not recall time since last screening test

### Acceptability of HPV self-sampling

HPV self-sampling acceptability is shown using both qualitative (Table 2) and quantitative (Table 3) data. Table 2 summarises responses by the seven constructs identified in the theoretical framework of acceptability proposed by Sekhon et al. (2017) [44]: intervention coherence, affective attitudes, self-efficacy, perceived effectiveness, opportunity costs, burden and ethical concerns. The definition of each acceptability construct is shown together with selected quotes from participants. Table 3 shows the results of the questionnaires which were completed after the participants trialled each device. Overall, 40 women trialled at least one device (Table 1).

#### Intervention coherence

Although most participants reported having undergone CC screening (72%, Table 1), qualitative results revealed that knowledge about HPV, CC and CC screening was generally lacking. Most were unaware that the screening test aimed to detect HPV infection, precancerous cervical lesions or even CC. Many participants believed that the test was part of routine pregnancy check-ups as cytology was often offered opportunistically during pregnancy, and two participants even believed that the test was to check on their babies. Overall, women reported receiving limited information and felt that doctors over-simplified their explanations about the purpose of the test: “*They just told me that it’s a women’s health control to check that everything is ok” (SSI MC04*,* older (≥ 40 years old) Moroccan woman)*.

Women often referred CC as “*uterus cancer*” or as a cancer in the “*women’s intimate zone*”, “*women’s inner parts*” or simply “*down there*”. They perceived CC as a fatal or difficult to cure disease, associating it with pain, and believing that its treatment could cause infertility. Some women also linked CC to “*lack of hygiene*”, “*hormonal problems*” and considered it more common after menopause or in older ages.

Most participants were unfamiliar with HPV and unaware of its connection to CC. Those who knew about the virus had either been diagnosed with an HPV infection or encountered this term through their daughters’ school-based vaccination programmes. However, they reported not having information about HPV transmission, except a few women who linked CC to “*multiple sexual relationships*”.

After being informed that HPV is sexually transmitted, some women drew comparisons to other STIs, such as HIV. Our Moroccan and Pakistani participants showed a notably low perception of STI risks, believing that HPV is less prevalent in their communities compared to native European populations. They attributed this to their cultural and religious values, such as virginity before marriage and having monogamous relationships, which they believed protected them from STIs. However, a few women from both communities questioned this belief, suggesting that some men’s sexual behaviours could put their wives’ health at risk (see illustrative quotes in Table 2). None of the participants knew that HPV could remain asymptomatic and be transmitted after many years. They also believed that HPV is only transmitted through sexual intercourse with penetration, potentially diminishing their risk perception. A young Moroccan participant, who self-identified as a lesbian, remarked: *“So*,* yes*,* after what you are telling me now [that HPV can also be transmitted through genital contact]*,* I probably have it [the virus]*,* yes” (SSI MC08*,* younger (< 40 years old) Moroccan woman)*.

Overall, women from both cohorts had never heard about HPV self-sampling and they seemed not to share the same risk perception towards HPV infection as to CC. They perceived CC as a frightening disease that every woman is at risk of, but HPV infection was not considered a serious health treat. This misconception led them to express preference for cytology over a test to detect HPV: *“In my community*,* I think women would prefer to undergo the test to detect cancer instead of the one to detect the virus*,* because it (CC) scares them more” (SSI MC11*,* younger Moroccan woman)*.

**Table 2:** Qualitative findings on HPV self-sampling acceptability organised by the theoretical framework of acceptability components proposed by Sekhon et al. (2017)

Barriers & facilitators	Excerpts from FGD & SSI	
**Construct 1 - Intervention coherence**: ***the extent to which the participant understands the intervention and how it works***		
• Limited knowledge about cervical cancer and HPV	*“My cousin told me a few days ago that this type of cancer exists*,* before I didn’t know about it” (SSI PC02*,* younger Pakistani woman).*	
• Inadequate information about the screening test and confusion about the purpose of the test	*“They only do this test when the woman is pregnant. If the woman is not pregnant*,* they don’t do this test (…) I did it and when I went to the doctor the next time*,* I asked for the test*,* and they told me that it’s not necessary” (SSI PC05*,* younger Pakistani woman).* *“I thought they were going to check my vagina and when they introduced the stick inside*,* I thought they were taking a sample to check about my baby*,* that’s it” (SSI PC02*,* younger Pakistani participants).*	
• Misconceptions about risk to cervical cancer and HPV	*“In my case*,* the probability to get this virus would be very low because*,* as I said*,* we are Muslim*,* and we only have this type of intimate contact with only one person in our entire life” (SSI PC04*,* older Pakistani woman).* *“The husband leaves God’s pathway and does something with another woman; then he comes back home ‘dirty’ and sleeps with his wife*,* and the poor woman doesn’t know anything*,* and he passed his ‘dirt’ [virus] to her until it becomes cancer” (FGD 1*,* older Moroccan woman).* *“In theory we all have to stay virgins before marriage*,* but I know about men who live here [in Spain] and they are not married and live with Latin women and then they go to my country [Pakistan] to get married” (SSI PC05*,* younger Pakistani woman).*	
**Construct 2 - Affective attitudes**: ***how an individual feels about taking part in an intervention***		
• Preference for clinician-based over self-sampling	*“I prefer to go to the gynaecologist because I will be 100% sure that the test is done correctly and the result is right” (SSI PC01*,* younger Pakistani woman).* *“Personally*,* I would be afraid. I wouldn’t dare to do it (self-sampling)*,* I won’t know how to do it*,* I prefer to go to the doctor” (FGD 3*,* younger Moroccan woman).*	
• Overcoming barriers related to shyness and offers privacy	*“Some people feel shyness in front of doctors so that’s the advantage of doing it at home” (FGD 3*,* younger Pakistani woman).*	
• Convenient, comfortable, and faster	*“I prefer to do it at home*,* you are more comfortable*,* you can do it without feeling embarrassed and you can save time. It’s something we can do it*,* it’s not difficult” (FGD 3*,* older Moroccan woman).*	
• Overcoming barriers related to access health services (e.g. difficulties to get appointment)	*“It’s much better to do it at home*,* you don’t need an appointment with the doctor… This kit is the solution for all the problems! I don’t have to explain so many times at the reception of the CAP (primary health centre) the reasons for the appointment with the gynaecologist… This is perfect*,* even if I need to buy it*,* it’s alright*,* I will pay*,* and everyone would pay” (SSI PC05*,* younger Pakistani woman).*	
**Construct 3 - Self-efficacy**: ***the participants’ confidence that they can perform the behaviour(s) required to participate in the intervention***		
• Lack of confidence to perform self-sampling correctly	*“What if I don’t get the sample correctly and I keep myself with the doubt? That’s why I say that I’ll be sure that the sample has been collected correctly with the doctor” (FGD 0*,* older Moroccan woman).* *“It would take me hours only to think about it*,* whether to do it or not. I think it’s difficult to do it by myself. I would ask my daughter whether I have done it correctly or not”(FGD1*,* older Pakistani woman)*	
• Fear to harm themselves	*“People like me who don’t trust themselves in things like this (self-testing) will feel insecure of not doing it correctly and harming themselves or feeling pain” (SSI PC06*,* younger Pakistani woman)*	
• Low literacy, specifically about female anatomy	*“European women*,* obviously*,* they will know how to use it (self-sampling test)*,* but for Moroccans*,* most of Moroccans who live here (Spain) are from rural areas… I don’t think they’ll know how to use it. Many don’t have studies; I don’t think they’ll know” (FGD 0*,* older Moroccan woman).* *“I think due to lack of education*,* they won’t perform it correctly*,* which will affect the test results as well. It won’t have any advantage of doing it at home” (FGD 0*,* younger Pakistani woman).* *“I would go to the gynaecologist because I’m afraid of doing it by myself. I cannot read and I wouldn’t understand anything” (SSI PC11*,* older Pakistani woman).*	
• Limited experience with tampons and vaginal products	*“I’m very afraid of touching this intimate area*,* honestly. I’ve never been able to use a tampon. I have tampons and I would like to use them*,* but I couldn’t. I am worried about this area*,* I need to care it very well*,* using quality pads*,* changing the panty. I cannot introduce anything in this area [vagina]*,* you know? (…) I’m scared of introducing something and it’ll stay inside*,* and I cannot take it out. Then*,* I had to go to the doctor… Honestly*,* I never was able to use a tampon” (SSI MC04*,* older Moroccan woman).*	
**Construct 4 - Perceived effectiveness** ***(the extent to which the intervention is perceived as likely to achieve its purpose)***		
• Distrust the test result due to bad experiences with COVID self-tests	*“These tests have advantages … and disadvantages as well. My brother who is a doctor brought the kit to test for COVID and the result was positive and the second day when he bought it from the pharmacy it was negative. Either the device had a problem or maybe he cured the second day from COVID. You are not sure about these devices” (FGD 0*,* younger Pakistani woman).* *“If it’s a reliable test*,* I mean that it really detects the virus*,* then I may do it [self-screen]. But if it’s like COVID tests*,* that sometimes detected the virus and other times didn’t*,* I wouldn’t do it” (SSI MC11*,* younger Moroccan woman).*	
**Construct 5 - Opportunity costs** ***(the extent to which benefits, profits, or values must be given up to engage in an intervention)***		
• Reduced engagement with health providers and missing the identification of other SRH issues	*“If women can do this test by themselves at home*,* they will not go to the gynae anymore… and sometimes you go for one thing*,* and it turns out that you have other health issues” (SSI MC09*,* younger Moroccan woman).*	
**Construct 6 - Burden** ***(the perceived amount of effort that is required to participate in the intervention)***		
• Concerns about HPV positive test	*“And the day I will get the result… God! I’ll hope it’s ok. Imagine if the result is not good [HPV positive]*,* would you tell your husband?” (FGD 1*,* older Moroccan woman)*	
**Construct 7 - Ethical concerns** ***(the extent to which the intervention has good fit with an individual’s value system)***		
• Belief that screening can affect virginity	*“I’m still single*,* so I have never got screened. I cannot use this device as it can affect my virginity*,* you know?” (SSI MC11*,* younger Moroccan woman).*	

CC: Cervical cancer; FGD: Focus group discussion; HPV: Human papillomavirus; SSI: Semi-structured interview. Younger participants defined as < 40 and older defined as ≥ 40 years

#### Affective attitudes

The first reaction of many participants was to express a preference for health provider-based CC screening over self-sampling. Some women suggested that for a disease as serious as cancer, it would be safer to be screened by a doctor rather than themselves. By the end of the FGDs and SSIs and after a short demonstration of the HPV self-sampling devices, 56% participants (*N* = 40) accepted the invitation to take the devices home and tried at least one of the two self-sampling devices (Table 1). Among those, Moroccan women seemed to have a higher acceptance rate (71%), compared to Pakistani participants (43%) (*p* = 0.04). Furthermore, HPV self-sampling acceptability was slightly higher among never-screened (71%) compared to those screened previously (57%) (*p* = 0.4), although not statistically significant. Similarly, slightly higher acceptability was observed among employed (77%) compared to unemployed (52%) women (*p* = 0.1). No differences in acceptability were observed regarding the time since migration to Spain (56%; 52%, and 59%, for less than 5 years, 6 to 10 years and more than 10 years in Spain, respectively) (*p* = 0.9). However, recently migrated Moroccan women (less than 5 years in Spain) showed higher acceptability (80%) than Pakistani women (25%) (*p* = 0.05). Notably, all women who had never heard about CC screening refused to try the self-sampling devices.

**Fig. 1:**
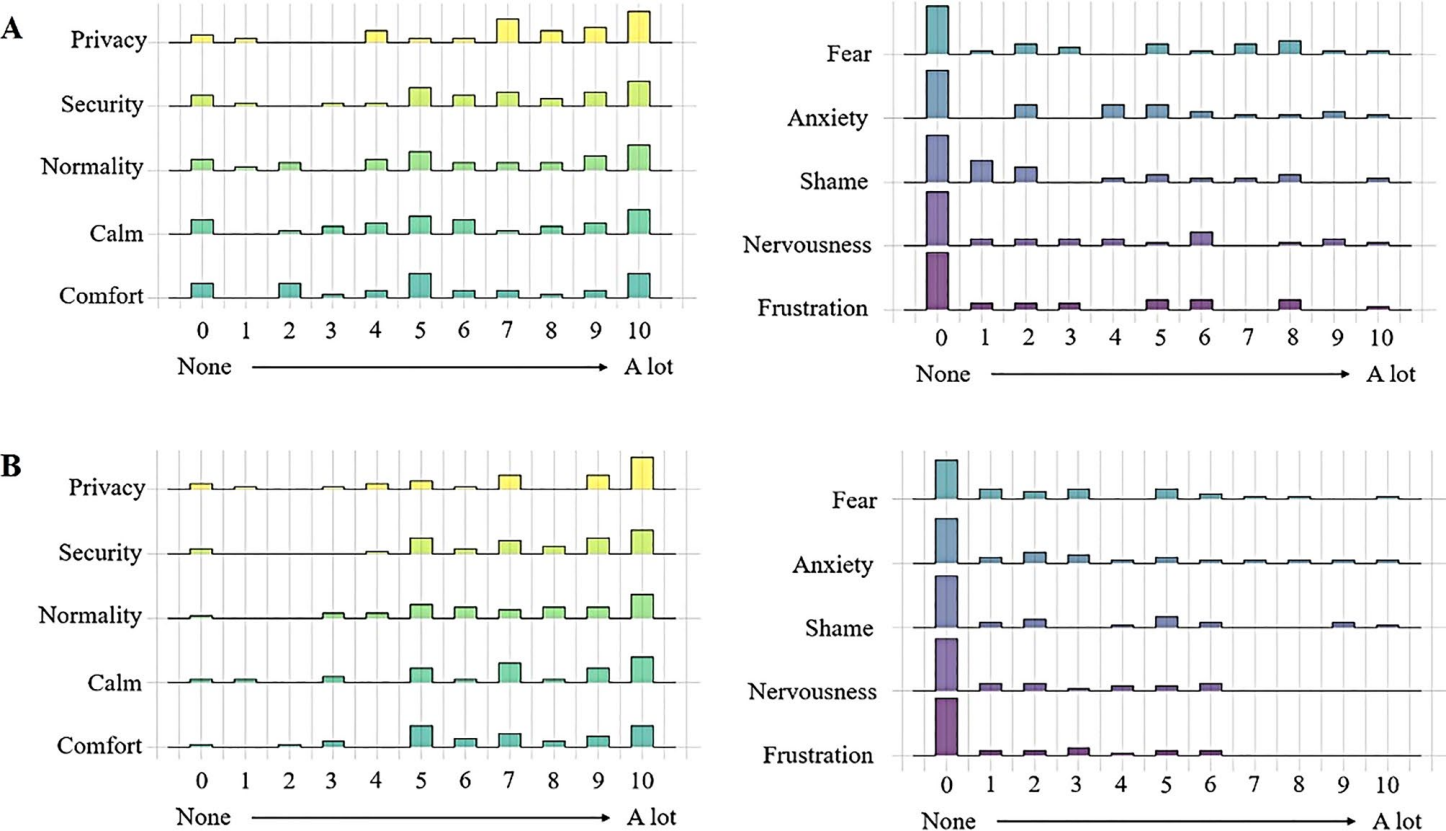
Density plots of feelings experienced by women when collecting sample with swab (**A**) and brush (**B**)

During the group discussions and interview sessions, study participants mentioned several benefits of HPV self-sampling. They acknowledged the opportunity that HPV self-sampling brings to overcome barriers, such as shyness and being examined by a male doctor. In addition to the privacy that self-sampling offers, women also commented that performing the test at home is more comfortable and convenient, especially for those women who have time constraints due to childcare responsibilities or full-time employment. Older participants (≥ 40 years old) referred more often to overcome shyness as the main motivator to use HPV self-sampling and younger participants (< 40 years old) from both cohorts emphasized that the main benefit of using self-sampling would be to remove accessibility barriers, in particular they mentioned that self-sampling would be a solution for the long waiting lists they often face in the Catalan health system to visit a specialist. The quantitative data regarding feelings after undergoing self-sampling corroborate some of these findings: reduced levels of shame and increased feelings of privacy and comfort (Fig. 1).

The preference for clinician-based CC screening remained after trialling the self-sampling devices: more women still favoured clinician-based screening than self-sampling regardless of the device tried (Table 3), though a higher proportion of participants indicated a preference towards self-sampling when using the FloqSwab^®^ Copan (40%, 15/38) compared to the Evalyn^®^ Brush (26%, 9/35) and the participants who tried the devices generally showed a positive attitude towards HPV self-sampling. The vast majority would like to use the self-sampling device again, would recommend this sample collection method, and were receptive to use it as part of the CC screening programme (> 90% of those using the FloqSwab, Table 3).

**Table 3:** Attitudes towards HPV self-sampling and usage experiences among those who accepted to try each device^1^

	FloqSwab^®^ Copan		Evalyn^®^ Brush	
	N	(%)^2^	N	(%)^2^
**Participants who accepted to use HPV self-sampling** ^4^	**38**	***(53.5%)*** ^***3***^	**35**	***(49.3%)*** ^***3***^
**What would you prefer**,** self-sampling or having the sample collected by a healthcare provider?**				
* Self-sampling*	13	*(34.2%)*	9	*(25.7%)*
* Healthcare provider-based*	15	*(39.5%)*	16	*(45.7%)*
* Both options are fine to me*	8	*(21.1%)*	7	*(20.0%)*
* None of them*	1	*(2.6%)*	1	*(2.9%)*
* I don’t know / Prefer not to answer*	0	*(0.0%)*	1	*(2.9%)*
**Do you think the self-sample has been collected** **properly?**				
* Yes*,* I’m sure*	21	*(55.3%)*	13	*(37.1%)*
* I’m not sure I picked it up right*	16	*(42.1%)*	17	*(48.5%)*
* I’m sure I picked it up wrong*	0	*(0.0%)*	2	*(5.7%)*
* I don’t know / Prefer not to answer*	0	*(0.0%)*	1	*(2.9%)*
**Would you trust the result of this self-sampling test?**				
* Yes*	19	*(50.0%)*	17	*(48.6%)*
* No*	11	*(28.9%)*	14	*(40.0%)*
* I don’t know / Prefer not to answer*	5	*(13.2%)*	2	*(5.8%)*
**Did you need any help to collect the self-sample or to understand the instructions?**				
* Yes*,* to collect the sample & understand the instructions*	1	*(2.6%)*	1	*(2.9%)*
* Yes*,* but only to understand the instructions*	11	*(28.9%)*	13	*(37.1%)*
* No*,* I’ve done it all by myself*	25	*(65.8%)*	20	*(57.1%)*
**What do you think about the use of the self-sampling?**				
* I found it very easy*,* easy and simple to use*	27	*(71%)*	22	*(62.8%)*
* Normal*,* neither difficult nor very easy*	11	*(28.9%)*	7	*(20%)*
* I found it very difficult or difficult to use*	0	*(0.0%)*	5	*(14.3%)*
**Do you think the self-sample device is safe?**				
* Yes*	35	*(92.1%)*	25	*(71.4%)*
* No*	1	*(2.6%)*	6	*(17.1%)*
* I don’t know / Prefer not to answer*	1	*(2.6%)*	4	*(11.5%)*
**Would you use self-sampling again?**				
* Yes*	36	*(94.7%)*	29	*(82.8%)*
* No*	0	*(0.0%)*	3	*(8.6%)*
* I don’t know / Prefer not to answer*	1	*(2.6%)*	1	*(2.8%)*
**Would you recommend self-sampling to a family ** **or a friend** **?**				
* Yes*	36	*(94.7%)*	29	*(82.9%)*
* No*	0	*(0.0%)*	3	*(8.6%)*
* I don’t know / Prefer not to answer*	1	*(2.6%)*	1	*(2.9%)*
**Would you like self-sampling to be used as a future screening method?**				
* Yes*	35	*(92.1%)*	27	*(77.1%)*
* No*	1	*(2.6%)*	4	*(11.4%)*
* I don’t know/ Prefer not to answer*	1	*(2.6%)*	2	*(5.7%)*

^1^ Due to missing data, the total percentages may not add up to 100%. Note that participants who tried both devices (*n* = 33) appear twice in the table, but may have answered differently for each device

^2^ Percentages correspond to column percentage

^3^ This percentage was calculated according to the total number of self-sampling acceptability study participants

^4^ Number of participants who tried the devices was calculated based on the total study participants (*N* = 71)

#### Self-efficacy

The main concern among participants regarding HPV self-sampling was the lack of confidence in their ability to correctly perform the sample collection. Prior to trying the self-sampling devices, almost half of the participants (45%, Table 3) reported to be worried about not doing it correctly, whereas 42% felt confident as long as they had adequate instructions (Data not shown). Contrary to initial perceptions exposed in the FGD and SSI (Table 2), more than half of the participants who tried the self-sampling devices felt confident (55%), but still 42% of participants reported to feel unconfident self-collecting the sample (Table 3).

This lack of confidence led some women to express fear of harming themselves and to believe that a health provider-based screening is safer and yields more reliable results (Table 2). Quantitative data from the non-acceptability questionnaire revealed the main reasons for rejecting self-sampling devices among all participants were fear of collecting a vaginal sample by themselves (39% and 37% for swab and brush, respectively) and preference for clinician-collected sample (22% and 18% for swab and brush, respectively) (Data not shown).

Some participants attributed this lack of confidence to low literacy and language barriers. For example, an older (≥ 40-year-old) Moroccan woman expressed concerns about difficulties that women from rural areas and with low literacy may face understanding the self-sample instructions and self-sampling themselves. Quantitative data confirmed that around 60% of participants reported to be able to understand the instructions and collect the sample without help (66% and 57% for swab and brush, respectively), but still around 30% of women reported needing help to understand the self-sampling instructions, which were only in Spanish (Table 3). Paradoxically, some participants with university studies and a good command of Spanish also reported not feeling confident about introducing any device in the vagina. For instance, an older (≥ 40-year-old) Moroccan woman argued that she never was able to use a tampon (Table 2).

Overall, women who tried the self-sampling devices considered them safe and easy to use when evaluated quantitatively (Table 3). Women expressed a more positive response towards FLOQSwab device compared to EvalynBrush^®^ device but given the limited number of participants in our study and the focus on assessing overall acceptability, formal statistical comparisons between devices were not performed, limiting any statistical conclusions.

#### Perceived effectiveness

Half of women who tried the devices expressed confidence in the results obtained from self-collected samples however around 30% had still doubted the accuracy of the test results (29% and 40% for swab and brush, respectively) (Table 3). Distrust in the test result was not only motivated by lack of confidence in collecting the sample properly, but also by a broader lack of trust in self-sampling devices influenced by negative experiences with COVID-19 self-tests (Table 2). These experiences heightened concerns about potential false negative results and the subsequent implications of delayed detection of an HPV infection.

#### Opportunity costs

Several women raised concerns that opting for self-sampling over clinician-based sampling might result in missed opportunities to check for other SRH issues, such as infections. They emphasized the importance of being able to ask healthcare providers (nurses, midwives and/or gynaecologists) questions about other concerns for instance vaginal pain, contraception or pregnancy termination services, which women generally find difficult to book medical appointments for. A Moroccan participant noted that gynaecology attendance might decrease if women self-collect their sample for CC screening, potentially affecting overall health.

Another participant suggested that undergoing sample collection by themselves at home might not be as safe as in the health centre given enhanced hygiene practices used by health professionals, such as wearing gloves.

#### Burden

A few women expressed concerns that a positive HPV test may have a negative impact on their marital relationship, suggesting a psychological burden. Despite the connection of CC with a STI and its potential stigma, Moroccan and Pakistani women perceived that HPV self-sampling would be positively accepted within their communities, including their husbands, if accurate and clear information about the risks and causes of CC, as well as the preventive purpose of the screening test were provided. However, a Moroccan participant pointed out that some husbands would accept the self-sampling test only because it will prevent their wives from being examined by a male doctor.

#### Ethical concerns

Concerns regarding how CC screening (cytology or HPV self-sampling) could affect virginity were raised multiple times. For instance, a young Moroccan woman explicitly declined the invitation of using the self-sampling devices due to her belief that screening could tear the hymen and result in the loss of virginity (Table 2).

### Enhancing HPV self-sampling implementation: women’s insights

#### Raising awareness about CC and HPV

Women suggested that the most effective strategy to raise awareness of a CC screening programme would involve listening to testimonies from women within their own community who had experience of using HPV self-sampling, having HPV infection, cervical precancerous lesions or even cancer. Moroccan participants identified schools as ideal venues to reach immigrant women, as these women regularly take their children to school and could attend informative sessions and interact with other mothers. Pakistani women proposed community and religious centres, especially mosques, as convenient locations for short health education sessions, as they are regular places for meeting and interacting with other mothers. Additionally, educational activities organised by non-governmental organizations (e.g. Spanish/Catalan language classes) were highlighted as popular among immigrant women. Others pointed out the importance of engaging men in health education sessions to enhance the prevention of HPV and CC, recognizing the role that men play in women’s health decisions and practices.

#### CC screening invitation and delivery of HPV self-sampling devices

Most Pakistani participants expressed a preference for being informed about CC screening and HPV self-sampling by SMS. While Moroccan women also mentioned SMS, the majority preferred receiving information through letter or phone calls. They expressed concerns that SMS might not convey sufficient information and could be easily overlooked. Additionally, some participants in both groups considered that an in-person invitation from a health professional during a visit for some other health reason would be more effective than letters or SMS. Others emphasized that a group-based invitation during an informative talk or workshop would be the most effective approach:

*“Personally I prefer a talk with other women*,* because each woman has her own experience and we can learn from each other”*,* (SSI MC04*,* older Moroccan woman)*

*“I think it should be through workshops as it’s easier to understand and you come to know about other women’s opinions as well”*,* (FGD 2*,* younger Pakistani woman)*

Regarding the distribution of HPV self-sampling devices, pharmacies and health facilities were preferred options for both Moroccan and Pakistani participants. Pharmacies were seen as accessible and convenient locations, whereas healthcare facilities were perceived as more reliable due to their ability to ensure confidentiality. Both locations were valued for allowing women to receive instructions from healthcare providers and to clarify any doubts they might have.

#### Health professional and peer support during self-sampling

Respondents suggested that confidence in correctly performing the self-sampling test could be increased by the presence of a health professional or a person with the same cultural and linguistic background from their own community who had been adequately trained to explain the procedure. This support, even if provided at a women’s home, was seen as reassuring. For instance, a few participants expressed a preference for performing the self-sampling with the assistance of their daughters, sisters, or someone in their own community, as it would make them feel more comfortable and confident.

Women also emphasized the importance of oral explanations and visual self-sampling instructions to overcome literacy and language barriers. Both groups proposed creating a didactic video in their local languages, offering detailed visual explanation of the self-sampling procedure. Moroccan participants proposed disseminating the video through community workshops and local TV channels. In contrast, many Pakistani women preferred accessing the video on their own mobile phones through WhatsApp or similar platforms. Younger participants also suggested the potential of social media platforms, such as Facebook, Instagram and Tik-Tok for distributing information and educational content.

## Discussion

Our study is the first to examine HPV self-sampling acceptability among Moroccan and Pakistani women in Spain. We identified significant barriers to CC screening including lack of information and misconceptions about HPV risk, which led some women to consider CC screening as irrelevant. Overall, participants expressed a preference for clinician-based over self-sampling. Around half of the participants accepted to use HPV self-sampling and their experience was generally positive. However, concerns about performing the test incorrectly and distrust in the test result were raised, which need to be addressed prior to HPV self-sampling implementation among these two groups of women. Despite these concerns, women also described motivators for self-sampling and made some suggestions to enhance the newly organised population-based CC screening programme in Catalonia and to improve screening participation within their communities.

We observed HPV self-sampling acceptability varied based on country of origin, time since migration to Spain, employment status, and history of CC screening. The overall acceptability rate in our population (56%) differs from previous studies among Spanish-born women [40, 42] and migrants from Morocco and Pakistan in other settings [11, 53], which reported over 80% willingness to undergo self-sampling. By country of origin, Pakistani women appeared to show lower HPV self-sampling acceptability (43%) compared to Moroccan women (70%), which is consistent with previous research conducted with Pakistani communities in the UK [54]. This could be attributed to various factors. First, CC screening recommendations in the participants’ countries of origin are different. Pakistan lacks official national screening recommendations [55], whereas Morocco started opportunistic screening of women aged between 30 and 49 (the age range of most women in our study) in 2010 [56], which may affect women’s awareness about CC screening. This could also explain our findings of apparently higher acceptance of self-sampling among recently migrated Moroccan women compared to their Pakistani counterparts. Second, the long migration history of Moroccan women in Spain differs from those from Pakistan, who arrived in the country relatively recently which may affect accessibility barriers and their familiarity with the Catalan health system.

Our quantitative findings also indicated that immigrant women with formal or informal employment may have a higher acceptance of self-sampling compared to unemployed women, which may be due to higher social integration in the Catalan society and more access to host country health information. This finding also aligns with results from other Spanish studies, where employment, educational level and nationality have shown to be some of the main determinants of CC screening inequalities [57]. Similarly, it seems that the proportion of never-screened participants who took the opportunity to self-sample (71%) was slightly higher than those screened women (57%), despite being informed that they would not receive the test result. This supports the evidence that motivation to take part in screening may be higher in never- and over-due screened women when they are offered a choice [36], in this case, through community health workers, and when they feel that self-sampling helps them to overcome clinician-based screening barriers, such as shyness or discomfort [30].

Main barriers for HPV self-sampling were lack of information about the purpose of the screening test, low risk perception and self-efficacy concerns. Women showed limited knowledge about CC and its connection with HPV, and the available screening services. Many women were unaware of the purpose of the test and some confused it with routine pregnancy check-ups, potentially undermining regular participation in CC screening programmes. Lack of knowledge and confusion about the purpose of the test have been previously reported in some studies among migrants [58], however in other studies immigrant women knew about the benefits of the test [59]. However another study from England showed that the level of awareness of CC screening varied according to the country of origin [60]. Participants generally perceived their risk of HPV infection as low, as they reported not engaging in pre-marital sex or having multiple sexual partners, similar to findings in Canadian Muslim women [61], leading some of them to believe that screening was irrelevant within their communities.

This raises the need for more accurate, comprehensive, and culturally and linguistically appropriate information -such as materials and initiatives that are co-designed with the target population and tailored to their age, language, ethnicity, gender identity and culture, including religion- which is recognized as a vital component to improve CC screening uptake among under-screened women [62]. Such information addresses specific barriers and facilitators to participation, uses empathetic and simple communication, and respects cultural sensitivities ensuring *‘intervention coherence’*, so participants understand the importance of CC prevention, how HPV is transmitted and how CC screening programmes work, including HPV self-sampling.

Another important barrier that could affect participation in CC screening using HPV self-sampling was lack of confidence in collecting the sample correctly. In our study, around half of participants lacked confidence to collect the sample correctly –only 42% felt confident pre-trial, rising to over 50% among those who tried post-trial. This is consistent with a substantial body of literature that points to low *‘self-efficacy’* as one of the major barriers for HPV self-sampling to be successfully implemented among minority ethnic groups [11, 38, 63–65]. This low perceived self-efficacy in performing self-sampling correctly led participants to express concerns about self-harm and distrust the test result. Overall, Moroccan and Pakistani women seemed to feel more confident using swab-based than brush-based vaginal devices, as seen in previous research [18].

Language barriers, low literacy and an apparent lack of experience using vaginal menstrual hygiene products, such as tampons, may explain this low self-efficacy perception among Moroccan and Pakistani women. History of tampon use was associated with negative perceptions of physician-led screening, but not for self-sampling among a study in Japanese women, and lack of tampon use was not a barrier against willingness to use self-sampling again [66]. In line with these findings, our participants who tried at least one self-sampling device showed a willingness to try it again. However, it is noteworthy that still around 30% of participants reported needing assistance to understand the instructions of the self-sampling devices provided only in Spanish, which is consistent with previous research on immigrant populations in other settings [61, 67].

Beyond the lack of confidence in self-collection, other factors might impact ‘*perceived effectiveness’* of HPV self-sampling. Several of our participants questioned the validity of self-sampling devices due to negative experiences with the accuracy of COVID 19 self-tests, which led them to distrust and perceive HPV self-sampling as an ineffective method for early detection of CC.

We identified the possibility of missing the chance of addressing other SRH issues during the screening visit (e.g. STIs, family planning) as the main ‘*opportunity cost*’, a concern also identified in a recent intervention study among immigrant women in Canada [67]. Other participants also noted that a screening programme primarily using self-sampling might decrease gynaecology attendance. Monitoring other services for potential impact and education to increase awareness of other concerns to encourage women to consult a healthcare provider must be a priority to increase reduce health disparities.

We also identified an *‘ethical’* concern regarding the use of HPV self-sampling among these populations. Participants, especially younger women, were concerned that the use of vaginal devices may interfere with virginity making self-sampling unacceptable for them. On the other hand, we did not find that HPV self-sampling incurs a substantial ‘*burden’.* Some women, particularly from the Moroccan cohort, raised concerns that a positive HPV test could negatively affect their marital relationships, potentially causing psychological distress. This concern was also identified in previous studies with immigrant women in the UK [18, 53], but also in autochthonous populations [68, 69].

Despite the above concerns, Moroccan and Pakistani women highlighted various positive ‘*affective attitudes’* about the use of HPV self-sampling, such as the comfort, privacy and convenience that this self-collection method offers, in line with previous research [24, 70]. Some women also mentioned that self-sampling could be a “solution” for the long waiting lists in the Catalan health system. In this sense, it is also crucial to acknowledge that self-sampling may also have the potential to overcome common health system accessibility barriers, such as lack of healthcare professionals.

Participants expressed different preferences for educational interventions regarding CC prevention and HPV self-sampling implementation. For instance, Moroccan participants preferred school-based interventions, whereas more Pakistani women favoured faith-based centres for awareness activities. Both groups indicated the need of getting support from family and peers from their communities to overcome self-efficacy barriers to HPV self-sampling, an approach shown to be effective in recent interventions in India [71]. The possibility of including males in education interventions to improve CC screening participation should also be evaluated due to the influential role that partners play in women’s health decision-making, as it was demonstrated in a previous study with these two groups of women [72]. Women in both groups also expressed a preference for co-producing intervention materials (e.g. videos) with members of their own communities.

The main strengths of our study include the engagement of a diverse sample of Moroccan and Pakistani immigrants living in Catalonia, Spain, who were able to express their views in their own languages. This was facilitated by the availability of moderators and interviewers with the same cultural and linguistic background as the participants, which effectively created an environment of trust and comfort during the conversations. Cultural and linguistic alignment is crucial for ensuring honest and open communication, which enriched the data collected. Additionally, the combination of qualitative and quantitative methods provided a more comprehensive understanding of Moroccan and Pakistani women’s perceptions and attitudes towards CC screening and, particularly, HPV self-sampling. While the qualitative methods (e.g. FGD, SSI) allowed us to explore anticipated reactions and intentions to use HPV self-sampling, the survey captured individual experiences and views on its use, as well as preferences regarding specific self-sampling devices. Our study’s context, set within the implementation of a CC population-based screening programme in Catalonia, adds another layer of relevance and applicability to our findings. The timing of our research means that the insights gained can directly inform and improve the implementation of the CC screening programme, potentially increasing its effectiveness and uptake among these immigrant communities.

It is important to note that the number of women included in the qualitative component of our study was substantial, however the quantitative sample size was relatively small, limiting the ability to draw firm conclusions. Another limitation is the use of convenience and purposive techniques for participant recruitment. These methods, while practical and often necessary in exploratory research, can introduce selection bias and limit the generalizability of the findings. In addition, we did not provide any test results back to the women, as self-samples were not processed for HPV detection, which may have discouraged some women from trying the devices. It is unclear how this may have impacted participation and how many women would have accepted if the test was offered as part of the regular Catalan screening programme. This is the first study conducted in Spain addressing HPV self-sampling acceptability -including knowledge, perceptions and attitudes- among Moroccan and Pakistani immigrant women and it provides invaluable insights on the health needs and expectations of Pakistani and Moroccan immigrant women, serving as a starting point for future implementation research aiming to tackle CC screening inequities.

## Conclusion

This study shows that efforts are still needed to raise awareness and to empower Moroccan and Pakistani women living in Catalonia, Spain, with accurate and culturally appropriate information about the importance and benefits of CC screening. Women expressed their preference for clinician-based screening over self-collection, but around half of the study participants were open to use HPV self-sampling and accepted the invitation to try self-sampling at home. However, many raised concerns regarding self-efficacy, which needs to be addressed to successfully implement an equitable population-based HPV self-sampling screening strategy. Tailored educational interventions, along with a community and/or peer-based approach appear to fit best to inform and educate women from Moroccan and Pakistani communities, foster confidence and advocate for the uptake of HPV self-sampling.

## Supplementary Information

Below is the link to the electronic supplementary material.

Supplementary Material 1

Supplementary Material 2

Supplementary Material 3

Supplementary Material 4

## Data Availability

No datasets were generated or analysed during the current study.

## Abbreviations

CC: Cervical cancer
LMIC: Low- and middle-income countries
FGD: Focus group discussion
HPV: Human papillomavirus
HIC: High income countries
RA: Research assistant
SMS: Short-message service
SRH: Sexual and reproductive health
SSI: Semi-structured interview
STI: Sexually transmitted infection
