# Supplementary material for: Moroccan and Pakistani women’s knowledge and perceptions on cervical cancer screening and HPV self-sampling acceptability in Catalonia, Spain: a mixed-methods study
Source: BMC Health Serv Res. 2025 Nov 20;25:1502. doi: 10.1186/s12913-025-13488-w (PMC12636231; doi:10.1186/s12913-025-13488-w)
Supplement: Supplementary file 1 — Supplementary Material 1 [file 12913_2025_13488_MOESM1_ESM.pdf]

INSTRUCTIONS for the moderators: How to use the FGD Guide:

The objective of this group interview is to explore barriers and facilitators to cervical cancer screening and acceptability of HPV self-sampling among Moroccan/Pakistani women living in Barcelona. In particular, we will be interested in asking questions about their knowledge, perceptions, beliefs and attitudes towards cervical cancer, as well as their prevention practices and experiences about cervical cancer screening. Additionally, we will ask some questions about their social lives and who are the people they trust for health information and advice.

- There are two levels of questions:

  Primary interview questions: appear in **the left column**. They address the topics that you as the moderator must ask and encourage participants to respond and discuss. ***The moderator's goal is to generate a maximum number of different ideas and opinions from as many different people.*** The discussion is free-flowing. Ideally, participant comments will stimulate and influence the thinking and sharing of others. The primary questions are suggestions for getting the discussion going and you do not need to read them verbatim. Instead, you can rephrase them. During the discussion of each topic, you can use the sub-questions/probing topics in the right column to stimulate the conversation and address these sub-topics in case participants do not mention them. You can and should formulate other questions to clarify or understand relevant comments during the discussion, for example, the 'whys'. It is important you to be engaged with the conversation, make all women feel comfortable and make sure all of them participate equally.

  Sub-questions/Probing topics: are in **the right column**. These are to assist and encourage further discussion with the participants in case they are providing little information. It's not a requirement to cover every probe. Which probes you may or may not ask will depend on what has already been discussed.

With permission from the participants, **Moderator 1** should take notes and these notes should be labelled with initials or participant's ID. You will take notes in a separate notes form or sheet, where you will also insert your initials, as well as the date, where the group interview is taking place and the start and end of the FGD. **Moderator 2** should facilitate the discussion.

Before starting the FGD, ensure the participants have provided written informed consent and before the end of the FGD ensure the participants filled in the sociodemographic questionnaire.

Moderator 1:
Moderator 2:
Date:
Place where the FGD takes place:
FGD Start Time:
FGD Stop Time:

FOCUS GROUP DISCUSSION GUIDE

INTRODUCTION
10 minutes

Thanks for coming today. Today's group interview has **two goals**: 1. to explore the challenges and facilitators you may face to access and use public health services in Barcelona and, in particular, cervical cancer screening program, and 2. to understand your preferences to undertake a vaginal smear (clinician vs self-sampling).

Before we start the discussion, we would like to give you a short introduction of the study and if you accept to participate in the focus group, we will ask your **written consent** and to sign off a confidentiality agreement. [*Oral explanation of the participant information sheet and study objectives – summary in page 1 of this guide*]

Please, read carefully the written consent form, as well as the confidentiality agreement, and sign them off. If you have any questions, feel free to ask. We will give a copy of all these documents for you to read them again, if you need it.

(…)

Thanks for accepting to participate in this study. I will give you now a short explanation on **how a focus group works.** This is an informal discussion, all views are welcome and it is expected to be an interactive session. If you have any question about this session and the research, please, you can ask now or at any time during the discussion.

There are only a few basic rules to keep in mind while participating today:

- Everyone is expected to be an active participant.
- There are no "right" or "wrong" answers.
- Speak freely but remember not to interrupt others while they are talking.
- Audio recording and note taking is for research purposes only and will be used for analysis. Names are not attached to the notes, instead we will use codes to ensure anonymity.
- All feedback today will remain confidential and anonymous. In order to maintain confidentiality, I just ask that anything that is said during our session is not repeated outside of our session

The session will have 2 parts with a little break to go to the toilet and we will also have some refreshments during the break, but you can help yourself tea or water during the session.

I would like to start this focus group with a brief round of **introductions**. We can start with the research team (…) . Now I will ask you to introduce yourselves and let us know how long have you been living in Spain/Catalonia**.**

EXPERIENCE WITH THE CATALAN PUBLIC HEALTH SYSTEM
5 minutes

**1. What is the perception of the Catalan health system within your community compared to the health system in your country?**	*-For example, how easy is to get an appointment with a doctor? Are there flexible schedules?*<br>*-How much satisfied are you with the health services provided? What would you improve?*<br>*- Are there specific barriers that women from Morocco/Pakistan face when they go to the doctor here in Barcelona?*

PERCEPTIONS AND ATTITUDES ABOUT CANCER IN GENERAL
5 minutes

<u>Vignette 1</u> **Fatima is a 41 year old woman, from Morocco/Pakistan who has been living in Barcelona for more than 10 years. She is married and she has 3 children aged 8, 10 and 11 years old. She was diagnosed with cancer three months ago.**

**2. With whom do you think Fatima should share her news about her disease and how do you think people in her community will react and feel when they learn about it?**	*-What do people in their community know about cancer?*<br>*-Will people in her community talk naturally about cancer or will prefer to avoid this type of conversations? Why?*<br>*-In which context do you think Fatima and the people in her community will talk about cancer-related topics?*<br>*-¿What do you know about cancer?*

KNOWLEDGE, PERCEPTIONS AND ATTITUDES ABOUT CERVICAL CANCER
15 minutes

Now, we are going to focus on our reproductive and genital organs. To do so, we are going to do a quick exercise. I will give you a page with a picture of the female reproductive system and you will need to place the right number in each of the 5 parts of our reproductive system (vagina, ovary, Fallopian tube, uterus, cervix). (See material 1)
(…)

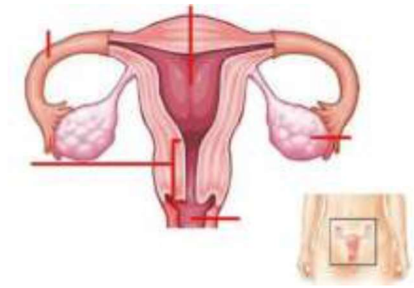

Now we are going to share where we placed each of the parts of the **female reproductive system**. Can someone tell us where is the cervix? *(Ask about each part)*

<u>Vignette 2</u> **Fatima was diagnosed with cervical cancer.**

**3. What do you know about this type of cancer?**	*- Where and what did you hear about this type of cancer?*<br>*-Causes and symptoms? Is a fatal or curable disease? How we can prevent it?*
**4. Do you think there is something that can make a woman, like Fatima, be at higher risk to developing this cancer than other women?**	*-Age*<br>*-Smoking*<br>*-Sexual activity *<u>Ask about HPV and its association with cervical cancer</u>*<br>*-Have you ever heard about this virus? Do you know someone who has had this virus?*<br>*-Do you know how this virus is transmitted?*<br>*-Do you think it is transmitted through sexual relationships?*<br>*- Do you think it is a sexual transmitted infection?*<br>*-What do you think about the link between cervical cancer and a sexually transmitted infection?*

**5. What do you think are the main concerns of Fatima about her cervical cancer?**	*Some examples:*<br>*-Fear to death*<br>*-Dependency of her children*<br>*-Stigma related to cancer*<br>*-Stigma related to infertility*<br>*-Side effects due to the treatment/disability*
**6. What do you think Fatima could have done to prevent her cervical cancer?**	*Some examples:*<br>*-Get screened for cervical cancer.*<br>*-Go to the doctor once she had the first symptom.*<br>*-Use protection in her sexual relationships.*<br>*-etc.*
**KNOWLEDGE, PERCEPTIONS AND EXPERIENCES ABOUT CERVICAL CANCER SCREENING**<br>**15 minutes**	
<u>Vignette 3</u> **Fatima felt healthy. He had no obvious symptoms. After having her last child 8 years ago, she did not visit a gynaecologist again, nor did she have a cytology.**	
**7. When and why do women like Fatima and you usually go to the gynaecologist or midwife?**	*Some examples:*<br>*Pre- and post- natal care; fertility problems; family planning, etc.*
**8. Could you tell me what is a cytology and why it is done and to whom?**	*-Can someone give us details on how is the procedure of this test?*<br>*-Why is it important? For whom?*
**9. Do you know other women like Fatima who have ever undertaken a cytology here in Catalonia or in your country? And you? Why did you do it and how was your experience?**	*-Was it painful, embarrassing? Didn't you understand for what the doctor was doing that test?*<br>*-Any difference between your experience in Spain and in your country?*
**10. To what extend do you think Fatima's religion might have influenced the fact that she did not go to a gynaecologist appointment in such a long time and did not undertake any check-up?**	*-Do you think her religion might prevent her to be attended by a male gynaecologist?*<br>*-Do you think her religion might prevent her to undertake specific tests or vaginal explorations?*
**BARRIERS & FACILITATORS TO CERVICAL CANCER SCREENING**<br>**20 minutes**	
We are going to finish this first part of the discussion before the break talking about the barriers that might prevent women like Fatima to attend a gynaecologist appointment.<br><br>To do so we will participate in a practical exercise. We are going to try to respond all together the following question:<br><br>***Why do you think Fatima did not go to the gynaecologist for such a long time and she did not undertake a cytology or got screened for cervical cancer****?*<br><br>We will have 5 minutes to write in post-its your own ideas about why a women like you might not go to a gynaecologist appointment to get screen for cervical cancer or undertake a cytology. We will paste the post-its on the flip-chart.	

Once we have all your ideas on the flip-chart, I will give you x cards with different statements describing an attitudinal or a barrier that might prevent you from going to gynaecologist appointment and to cervical cancer screening, in particular. The statements will include your ideas and new ones.

Once you had the cards, you will be asked to rank each of the barriers to cervical cancer screening, piling them according to their relevance based on your opinion and experience, and lining up the cards from most to least relevant. You will have three piles: 1. “Very relevant”, 2. “Less relevant” 3. “No relevant”.

1. **I never heard about cervical cancer and/or screening** (Hilverda et al 2021; Chan and So 2017, Vahabi and Lofters 2016; Addawe et al 2018; Gele et al 2017)
2. **I don’t really have time to do this test; I have other priorities, such as taking care of my children and work** (Addawe et al 2018 ; Grandahl et al 2012)
3. **I’d rather not know if I have cancer** (Marlow et al 2015, Hamdiui et al 2022, Addawe et al 2018)
4. **I have not symptoms; I don’t need to be screened** (Marlow et al 2015, Addawe et al 2018; (Gele et al 2017; Raymond et al 2014)
5. **I am not at risk to cervical cancer as I am in a marital relationship** (Chan et al 2019)
6. **I need to keep my virginity before I got married, so I cannot be screened (**Szarewski et al 2009; Addawe et al 2018)
7. **Cancer is God’s wish, there is nothing you can do** (Hamdiui et al 2021; Gele et al 2017)
8. **I would never go to an gynaecology examination with a male doctor; it is against my religion** (Hamdiui et al 2021; Hilverda et al 2021; Szarewski et al 2009, Vahabi and Lofters 2016; Addawe et al 2018)
9. **I am shy to do such test, it would be too embarrassing for me** (Hamdiui et al 2021; Hilverda et al 2021; Szarewski et al 2009, Vahabi and Lofters 2016; Addawe et al 2018)
10. **I am scared about gynaecological examinations; I heard it is painful** (Szarewski et al 2009; (Gele et al 2017; (Marlow et al 2015)
11. **I don’t participate in the screening, because I don’t understand the doctor’s language (**Addawe 2018, Grandahl et al 2012; Gele et al 2017)

11. These are the most relevant barriers that you have mentioned. What do you think about this barrier?

12. Is there anything we could do to overcome these important barriers?

BREAK (10 minutes)

BRIEF DEMONSTRATION OF HPV SELF-SAMPLING AND ITS USE
5 minutes

<u>Vignette 4</u> **After 8 years, Fatima visited the gynaecologist accompanied by her sister because she had some blood loss (vaginal bleeding). During the appointment, the doctor proposed her to undertake a test to detect the Human Papillomavirus, which is the necessary cause for cervical cancer. He offered her two options: collect the sample herself at home with a self-sampling device or have the sample collected by a health professional at the health centre.**

HPV SELF-SAMPLING ACCEPTABILITY AND SELF-EFFICACY
10 minutes

13. What do you think about undertaking a test to detect a virus which is transmitted through sexual relationships? How this test would be accepted in your community?

14. What do you think Fatima’s husband and people in her community may think about using this device for women to collect the sample themselves at home, instead of going to a health professional?

**INTENTION OF USING HPV SELF-SAMPLING; ADVANTAGES AND DISADVANTAGES AND SOCIAL SUPPORT NETWORKS**<br>**10 minutes**	
**15. Which screening method do you think Fatima chose? Why? (Optional – depending on timing)**	
**16. What are the advantages and disadvantages of each option offered to Fatima?**	
**17. How confident would you feel if you would decide to use the HPV self-sampling? Whom would you turn to for information and advice if you would need it?**	
**INVITATION TO CERVICAL CANCER SCREENING**<br>**5 minutes**	
**18. How would you like to be invited to a cervical cancer screening?**	*Some options:*<br>*-Through a letter posted to my home*<br>*-Text message*<br>*-WhatsApp or similar automated messages.*<br>*-In a community workshop with a health professional*<br>*-In the health centre, through a health profesional (my GP or midwife)*<br>*-Other ways (specify)*
**19. And if you would decide to use the HPV self-sampling, where would you like to receive and return it?**	*-For example, at home, in the pharmacy, in a health centre, in a community space, other ways (specify)*
**EXIT QUESTION**<br>**5 minutes**	
**<u>Vignette 5</u> Fatima decided to use the self-sample device. The test was positive (it seems that she had an infection caused by the Human papillomavirus) and a health professional performed a cytology to check if there was a lesion on the cervix. After various tests, Fatima was diagnosed with cervical cancer.**	
**20. Is there anything else that you would like to say about Fatima´s history and/or cervical cancer screening?**<br><br>- **Help participants to fill in the socio-demographic questionnaire.**<br><br>- **Offer and give participant the HPV self-sampling kits. Explain that it is a trial and they will not receive the test results, but you can get an appointment for them with the gyne, and give brief explanation of the acceptability questionnaire.**<br><br>Thank you very much for taking part in this study!	
