# Supplementary material for: Moroccan and Pakistani women’s knowledge and perceptions on cervical cancer screening and HPV self-sampling acceptability in Catalonia, Spain: a mixed-methods study
Source: BMC Health Serv Res. 2025 Nov 20;25:1502. doi: 10.1186/s12913-025-13488-w (PMC12636231; doi:10.1186/s12913-025-13488-w)
Supplement: Supplementary file 3 — Supplementary Material 3 [file 12913_2025_13488_MOESM3_ESM.pdf]

BARRIERS AND FACILITATORS TO CERVICAL CANCER SCREENING AND ACCEPTABILITY OF HPV SELF-SAMPLING AMONG IMMIGRANT WOMEN IN CATALONIA, SPAIN

FOCUS GROUP DISCUSSION - QUESTIONNAIRE

Date/Time ………………………….   Interview place: ……………………………………………

Participant ID: ………………………………………………………………………………

1. **What is your birth date?** (DD/MM/YYYY) _ _ / _ _ / _ _ _ _

2. **What year did you migrate to Spain?** ……………………………………………………………

3. **How many people do you live with?**
   I live alone ☐
   1 ☐
   2 ☐
   3 ☐
   4 ☐
   5 or more ☐
   Prefer not to answer ☐

4. **Which language(s) do you speak most often at home? *(You can tick more than one)***
   Spanish ☐
   Catalan ☐
   Arabic-Darija ☐
   Urdu ☐
   English ☐
   French ☐
   Other (specify) ☐ ________________________________
   Prefer not to anwer ☐

5. **What is your marital status?**
   Single ☐
   Married / Living with my partner ☐
   Separated ☐
   Divorced ☐
   Widowed ☐
   Other (specify) ☐ ________________________________
   Prefer not to answer ☐

6. **¿Do you have children? (If not, continue with question 8 ➡)**
   Yes ☐
   No ☐
   Prefer not to answer ☐

7. **How many children do you have?**
   1 ☐
   2 ☐
   3 ☐
   4 ☐
   5 or more ☐
   Prefer not to answer ☐

8. **Tick the box(es) in relation to your education *(You can tick more than one)***
   No academic school ☐
   Primary school ☐
   Secondary school ☐
   Vocational training ☐
   College – University degree ☐
   Koranic school ☐
   Other (specify) ☐ ________________________________
   Prefer not to answer ☐

9. **Are you employed?**
   Yes, I am employed (full-time, part-time, freelance) ☐
   No, I am unemployed (housewife) ☐
   Informal employment (without contract) ☐
   Retired ☐
   Prefer not to answer ☐

10. **Can you give an estimate of your annual household income?**
    Less than €12,000 ☐
    €12,001 – €20,000 ☐
    €20,001 – 30,000 ☐
    €30,001 – 40,000 ☐
    More than €40,001 ☐
    I don't know ☐
    Prefer not to answer ☐

11. **Do you have public health insurance in Catalonia?**
    Yes ☐
    No ☐
    Prefer not to answer ☐

12. **How well can you communicate at the health centre or hospital without the assistance of translators?**
    I always need a translator/mediator ☐
    Most of the times I need a translator/mediator ☐
    Sometimes I need a translator/mediator ☐
    I do not need a translator/mediator ☐
    Prefer not to answer ☐

13. **How often do you need someone to help you when you read instructions, pamphlets, or other written material from your doctor of pharmacy?**

Never ☐
Rarely / Sometimes ☐
Often ☐
Always ☐
Prefer not to answer ☐

14. **Do you know how to arrange an appointment with a gynaecologist in the public health system in Barcelona?**
    Yes ☐ No ☐ Prefer not to answer ☐

15. **If you have had an appointment with a gynaecologist, have you ever taken a cytology (Pap smear)?**
    Yes ☐ No ☐ I don't know ☐ Prefer not to answer ☐

16. **If you have taken a cytology (Pap smear), how long is it since the last time that you did so?**
    Less than 1 year ☐
    Between 1 and 2 years ☐
    Between 2 and 4 years ☐
    Between 3 and 5 years ☐
    More than 5 years ☐
    I don't know ☐
    Prefer no to answer ☐

17. **HPV self-sampling is a new method that allows women to do the cytology or get a vaginal sample themselves at home. If you were offered the chance to use the HPV self-sampling, would you take up the offer?**

    Yes ☐ No ☐ I don't know ☐ Prefer not to answer ☐

18. **How confident would you feel using HPV self-sampling?**
    I would worry that I had not done the test properly ☐
    I would feel very confident with adequate instructions ☐
    I don't know ☐
    Prefer not to answer ☐

19. **Would you say you are a religious person?**
    Very religious ☐
    Somehow religious ☐
    Very little religious ☐
    No religious at all ☐
    Prefer not to answer ☐

20. **Do you identify yourself as a Muslim person?**
    Yes ☐ No ☐ Prefer not to answer ☐

Thank you for your collaboration!
