# Supplementary material for: Moroccan and Pakistani women’s knowledge and perceptions on cervical cancer screening and HPV self-sampling acceptability in Catalonia, Spain: a mixed-methods study
Source: BMC Health Serv Res. 2025 Nov 20;25:1502. doi: 10.1186/s12913-025-13488-w (PMC12636231; doi:10.1186/s12913-025-13488-w)
Supplement: Supplementary file 4 — Supplementary Material 4 [file 12913_2025_13488_MOESM4_ESM.pdf]

HPV SELF-SAMPLING ACCEPTABILITY QUESTIONNAIRE

The following questions are to learn about your experience related to using the self-sampling device at home. We would appreciate it if you could answer all the questions below.

PARTICIPANT ID: ____________________________

1. Self-sampling performed on (dd/mm/yyyy): ____ / _____ / __________

2. How was the experience of collecting the vaginal sample using a self-sampling device?

- ☐ Very good
- ☐ Good
- ☐ Neither good nor bad
- ☐ Bad
- ☐ Very bad
- ☐ Other: ____
- ☐ I don't know/I prefer not to answer

3. Was it easy for you to understand the self-sampling instructions?

- ☐ Yes, they were very clear and easy to understand
- ☐ Quite clear and easy to understand
- ☐ Normal, neither so simple nor so complicated
- ☐ It was a bit difficult for me to understand
- ☐ It was very difficult for me to understand the instructions
- ☐ Other: ____
- ☐ I don't know/I prefer not to answer

4. What do you think about the use of the self-sampling device?

- ☐ I found it very easy and simple to use
- ☐ I found it easy to use
- ☐ Normal, neither difficult nor very easy
- ☐ I found it a bit complicated to use
- ☐ I found it very difficult to use
- ☐ Other: ____
- ☐ Don't know / prefer not to answer

5. Is the self-sampling device easy to introduce in the vagina?

- ☐ Yes, it enters very easily without any problem
- ☐ Yes, although it takes a bit of work
- ☐ Normal / indifferent
- ☐ It took me a while to enter
- ☐ It was quite difficult for me to introduce it.
- ☐ Other: ____
- ☐ I don't know / I prefer not to answer

6. Do you think the sample has been collected properly?

- ☐ Yes, I'm sure
- ☐ Yes, although with certain doubts
- ☐ I'm not sure I picked it up right
- ☐ I'm sure I picked it up wrong
- ☐ Other: ____
- ☐ I don't know / I prefer not to answer

7. Did you feel any pain while collecting the self-sampling?

- ☐ No, I haven't felt any pain
- ☐ No, but I felt some discomfort
- ☐ Yes, although not very intense
- ☐ Yes, an intense pain
- ☐ Other: ____
- ☐ I don't know / I prefer not to answer

8. What other feelings have you experienced when collecting the self-sample?
Mark the intensity of each feeling with an X, being 0-none and 10-a lot, or mark " I don't know " if you are not sure / know the answer.

	None 0	1	2	3	4	5	6	7	8	9	*A lot* 10	*I don't know*
Comfort												
Calmness												
Normality												
Safety												
Privacy												
Shame												
Fear												
Anxiety												
Frustration												
Nervousness												
Others: ________________												

9. How long did it take you to collect the self-sample?

- ☐ Less than 5 minutes
- ☐ Between 6 and 10 minutes
- ☐ Between 11 and 15 minutes
- ☐ More than 15 minutes

10. Did you need any help to collect the self-sample or to understand the instructions?

- ☐ Yes, but only to understand the instructions
- ☐ Yes, to understand the instructions and for sample collection
- ☐ No, I've done it all by myself
- ☐ Other: ____
- ☐ I don't know / I prefer not to answer

11. Do you think the self-sample is safe?

- ☐ Yes
- ☐ No
- ☐ Other:____________________________
- ☐ I don't know / I prefer not to answer

12. Do you think self-sampling will cause you any harm in the future?

- ☐ Yes
- ☐ No
- ☐ Other:____________________________
- ☐ I don't know / I prefer not to answer

13. Would you trust the result of this test performed by yourself instead of by a healthcare provider?

- ☐ Yes
- ☐ No *Why?* ______________________________________

- ☐ Other: ____________________
- ☐ I don't know / I prefer not to answer

14. What would you prefer, self-sampling (collecting the sample yourself) or having the sample collected by the healthcare professional?

- ☐ Myself
- ☐ The healthcare professional
- ☐ Both options are fine to me.
- ☐ None of them
- ☐ Other: ____________________
- ☐ I don't know / I prefer not to answer

15. If the self-sampling device will be used in the future, order from highest (1) to lowest (6) preference where would you like to pick up the self-sampling device? (1 = option you like the most, 6 = option you like the least)

- ☐ In a healthcare centre (primary healthcare centre, gynecology centre (ASSIR), referral hospital)
- ☐ In a pharmacy
- ☐ In a post office
- ☐ Have it sent/delivered to my home
- ☐ In a community facility *¿Which one?* ____________________
- ☐ Other: ____________________
- ☐ I don't know / I prefer not to answer

16. Order from highest (1) to lowest (5) preference where would you like to return the self-sampling device? (1 = option you like the most, 5 = option you like the least)

- ☐ In a healthcare centre (primary healthcare centre, gynecology centre (ASSIR), referral hospital)
- ☐ In a pharmacy
- ☐ In a post office or mail box
- ☐ In a community facility *¿Which one?* ____________________
- ☐ Other: ____________________
- ☐ I don't know / I prefer not to answer

17. Would you like self-sampling to be used as a screening test for early detection of cervical cancer in the future?

- ☐ Yes
- ☐ No
- ☐ Other: ____________________
- ☐ I don't know / I prefer not to answer

18. Would you use the self-sample again as a screening test to detect cervical cancer early in the future?

- ☐ Yes
- ☐ No
- ☐ Other: ____________________
- ☐ I don't know / I prefer not to answer

19. Would you recommend using self-sampling for cervical cancer screening to a family member/friend?

- ☐ Yes
- ☐ No
- ☐ Other: ____________________
- ☐ I don't know / I prefer not to answer

If you have any other comments you want to share, you can write them here:

____

Thank you very much for your contribution!

HPV SELF-SAMPLING NON-ACCEPTABILITY QUESTIONNAIRE

This questionnaire is to be completed by all women who participated in the study, but declined the invitation to try one or the two HPV self-sampling devices at home, or despite accepting the invitation and taking the devices home, they did not return the acceptability questionnaire within the expected timeframe (two weeks since they took the devices)
Those women who accepted the invitation to try the self-sampling devices, but did not return the acceptability questionnaire in two weeks, will be called by phone as a reminder and if they still want to participate in the trial, two more weeks will be given to return the acceptability questionnaire.

PARTICIPANT ID: ____________________________

1. Date (dd/mm/yyyy): ______ / _______ / ____________

2. Could you tell me the reason why you do not wish to participate in this trial?

- ☐ I have not time
- ☐ I am not interested in the study
- ☐ I prefer to go to a health centre or hospital and the sample to be taken by a doctor
- ☐ I am afraid of taking the sample by myself
- ☐ When I saw the device, it made me feel scared
- ☐ When I read the instructions, I found it difficult and I left it
- ☐ I think I will not be able to collect by myself the sample properly
- ☐ I found the device very big and I did not try it
- ☐ I prefer not to answer
- ☐ Other reasons ____

If you have any other comment that you wish to share, please, write it down here:
____

Thank you very much for your contribution!
